# Atorvastatin Improves Plaque Stability in ApoE-Knockout Mice by Regulating Chemokines and Chemokine Receptors

**DOI:** 10.1371/journal.pone.0097009

**Published:** 2014-05-09

**Authors:** Peng Nie, Dandan Li, Liuhua Hu, Shuxuan Jin, Ying Yu, Zhaohua Cai, Qin Shao, Jieyan Shen, Jing Yi, Hua Xiao, Linghong Shen, Ben He

**Affiliations:** 1 Department of Cardiology, Renji Hospital, School of Medicine, Shanghai Jiaotong University, Shanghai, China; 2 Department of Cell Biology, Key Laboratory of the Education Ministry for Cell Differentiation and Apoptosis, Institutes of Medical Sciencies, School of Medicine, Shanghai Jiaotong University, Shanghai, China; Brigham and Women's Hospital, Harvard Medical School, United States of America

## Abstract

It is well documented that statins protect atherosclerotic patients from inflammatory changes and plaque instability in coronary arteries. However, the underlying mechanisms are not fully understood. Using a previously established mouse model for vulnerable atherosclerotic plaque, we investigated the effect of atorvastatin (10 mg/kg/day) on plaque morphology. Atorvastatin did not lower plasma total cholesterol levels or affect plaque progression at this dosage; however, vulnerable plaque numbers were significantly reduced in the atorvastatin-treated group compared to control. Detailed examinations revealed that atorvastatin significantly decreased macrophage infiltration and subendothelial lipid deposition, reduced intimal collagen content, and elevated collagenase activity and expression of matrix metalloproteinases (MMPs). Because vascular inflammation is largely driven by changes in monocyte/macrophage numbers in the vessel wall, we speculated that the anti-inflammatory effect of atorvastatin may partially result from decreased monocyte recruitment to the endothelium. Further experiments showed that atorvastatin downregulated expression of the chemokines monocyte chemoattractant protein (MCP)-1, chemokine (C-X3-C motif) ligand 1 (CX3CL1) and their receptors CCR2 and, CX3CR1, which are mainly responsible for monocyte recruitment. In addition, levels of the plasma inflammatory markers C-reactive protein (CRP) and tumor necrosis factor (TNF)-α were also significantly decrease in atorvastatin-treated mice. Collectively, our results demonstrate that atorvastatin can improve plaque stability in mice independent of plasma cholesterol levels. Given the profound inhibition of macrophage infiltration into atherosclerotic plaques, we propose that statins may partly exert protective effects by modulating levels of chemokines and their receptors. These findings elucidate yet another atheroprotective mechanism of statins.

## Introduction

Statins are one of the first-line pharmacotherapeutic agents for hypercholesterolemia treatment in humans. In addition to decreasing low-density lipoprotein (LDL) cholesterol levels, numerous studies have reported that statins significantly protect atherosclerotic patients from inflammatory changes in coronary arteries [1].

During atherosclerosis initiation and progression, a common underlying cause of acute cardiovascular syndromes, such as myocardial infarction, is erosion or rupture of an unstable atherosclerotic plaque, which selectively increases circulating classical monocyte counts [2] and induces phenotypic changes that facilitate their migration into atherosclerotic lesions [3]. The inflammatory response continues as monocyte-derived macrophages, dendritic cells [4], and a subset of T cells migrate into the subendothelial area. We recently reported that atorvastatin suppressed the oxidative LDL-induced inflammatory response by inhibiting extracellular-signal-regulated kinase (ERK) phosphorylation, IκBα degradation, and cyclo-oxygenase-2 (COX-2) expression in murine macrophages. We also found that oxidative LDL-induced dendritic cell-like differentiation of macrophages was suppressed by atorvastatin through its effects on the p38 mitogen-activated protein kinase (MAPK) pathway [5]. Although some animal experiments have verified the effect of statins on plaque stability, little is known about the underlying in vivo mechanisms [6], [7].

Extravasation of monocyte-derived macrophages requires coordination among chemokines, selectins, and adhesion molecules [8]. Chemokines and their receptors play important regulatory roles in this process. With the discovery of monocyte subtypes, a new hypothesis has developed that adhesion molecule or chemokine receptor expression levels govern monocyte recruitment behavior. Indeed, it has been demonstrated that classical monocytes, which express higher levels of CCR2 (chemokine receptor-2) than non-classical monocytes, are less likely to be recruited to sites of inflammation in CCR2-deficient mice [9]. In addition to CCR2, CX3CR1 (CX3C chemokine receptor) is also important in coronary artery disease development [10]. These receptors play a critical role in the migration of monocytes and dendritic cells to atherosclerotic plaques, as well as in inflammatory activation during vulnerable plaque development.

Based on the existing evidence, we hypothesized that statins might improve plaque stability by regulating the expression of chemokines and their receptors. The apolipoprotein E (ApoE)-deficient mouse is a well-established genetic model in which advanced carotid artery lesions and plaque rupture can be induced through surgical ligation of two arteries, as we previously described [11]. In present study, 10 mg/kg/day atorvastatin was administered to ApoE-deficient mice after surgery. Expression levels of chemokines and their receptors on monocytes and macrophages were detected 8 weeks later, and parameters associated with plaque stability were also assessed.

## Results

### Atorvastatin Improves Plaque Stability without Decelerating Atherosclerosis in ApoE−/− Mice

**Figure 1:**
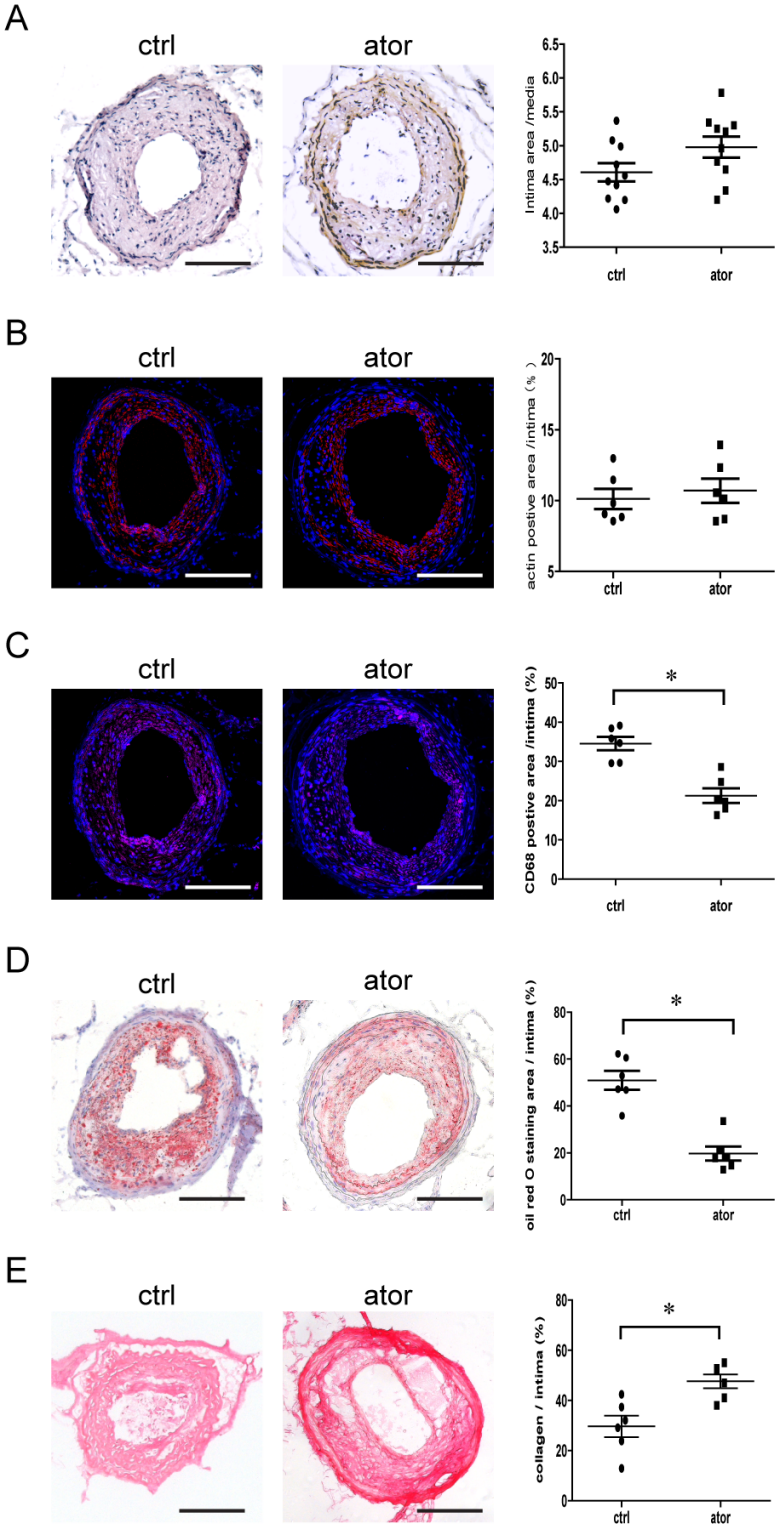
Effect of atorvastatin (10 mg/kg/d) on atherosclerotic plaque morphology in ApoE-/- mice. (A) Representative images of H&E staining (scale bar = 100 µm) and intimal surface/media ratio quantification in the control and atorvastatin-treated groups (*p*>0.05, n = 10). (B) Representative images of immunostaining for α-SMC actin (scale bar  = 100 µm) and quantification (*p*>0.05, n = 6). (C) Representative immunostaining for macrophages (scale bar  = 100 µm) and quantification (*p<0.05, n = 6). (D) Representative images of Oil Red O staining (scale bar  = 100 µm) and quantification (*p<0.05, n = 6). (E) Representative images of Sirius red staining (scale bar  = 100 µm) and quantification (*p<0.05, n = 6). Values are the mean ± SEM.

At 8 weeks after surgery, all of the ApoE−/− mice developed atherosclerotic lesions with vulnerable phenotypes in the LCCA as described before [11]. However, only 58.3% mice demonstrated vulnerable phenotype lesions in the atorvastatin-treated group (*p*<0.05), and they also exhibited decreased tendency for intraplaque hemorrhage (50% *vs*. 80%, *p*>0.05) and reduced incidence of vessel multilayer with discontinuity (25% *vs*. 70%, *p*>0.05) incidence compared with control group (Table 1). Plaque morphology was examined with histological analyses of serial sections. As shown in Figure 1A, atorvastatin-treated mice developed similar plaque areas with no difference in the intima-to-media area ratio compared to the control group (4.8±0.2 *vs*. 4.6±0.2, *p*>0.05). Similar results were observed for the mean vascular smooth muscle cell (SMC) area/intima surface area ratios (10.7±0.9% *vs*. 10.1±0.7%, *p*>0.05; Figure 1B). However, we detected significantly decreased macrophages/intima surface area ratio (21.3±1.9% *vs*. 34.6±1.7%, *p*<0.05; Figure 1C), Oil Red O staining intima surface area ratio (19.7±3.0% *vs*. 50.9±4.0%, *p*<0.05; Figure 1D), and collagen/intima surface area ratio (29.6±4.3% *vs*. 47.6±2.8%, *p*<0.05; Figure 1E) were detected in atorvastatin-treated mice.

**Table 1:** Lesion features.

	Stable phenotype	Vulnerable phenotype	Intra-plaque hemorrhage	Multi-layer with discontinuity
ctrl (n = 10)	0% (0)	100% (10)	80% (8)	70% (7)
ator (n = 12)	41.6%(5)*	58.3% (7)*	50% (6)	25% (3)

* *p*<0.05 *vs* group control.

### Atorvastatin Decreases Collagenase Activity of Atherosclerotic Plaque Lesions in ApoE−/− Mice

**Figure 2:**
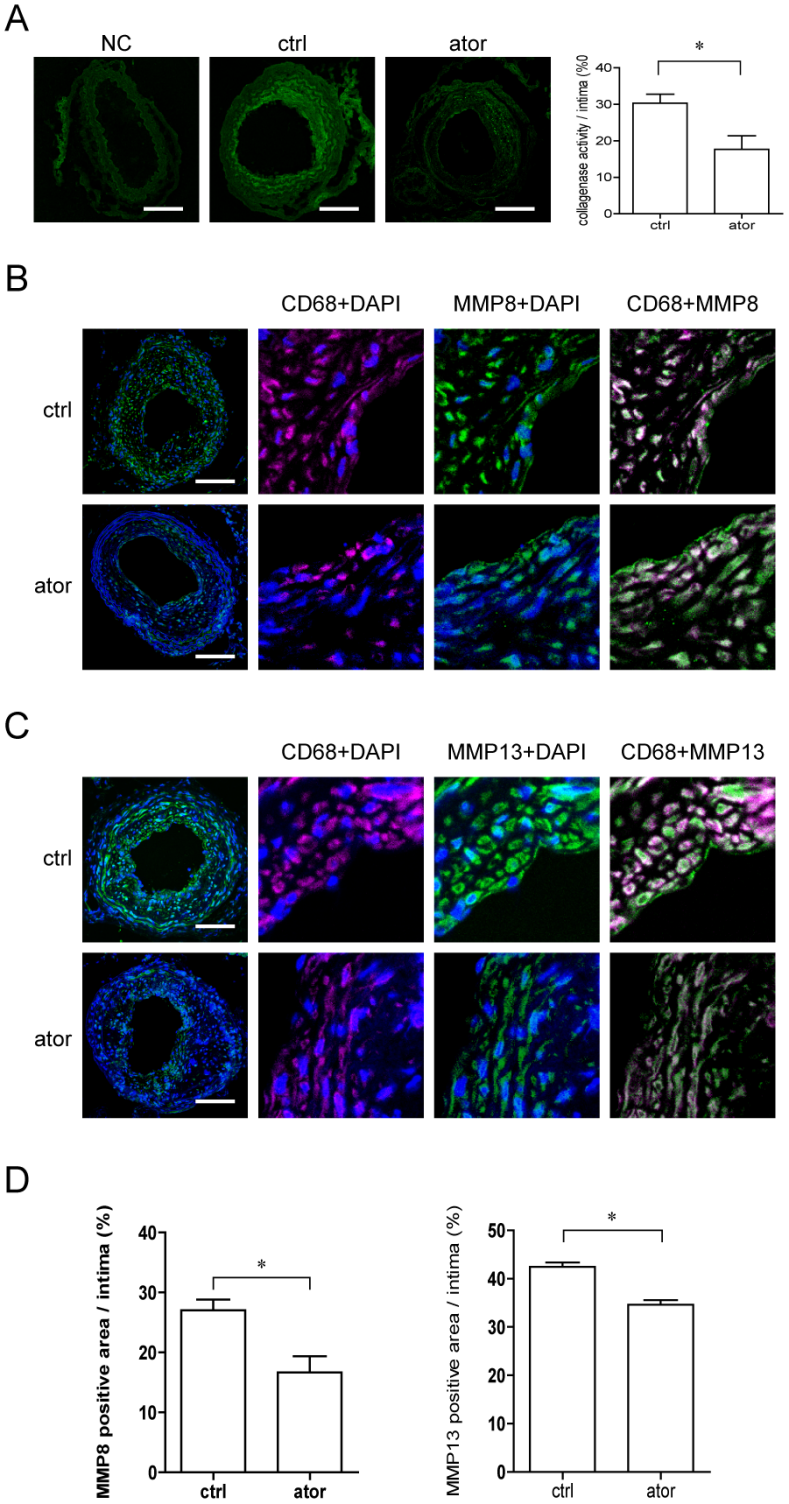
Effect of atorvastatin (10 mg/kg/d ) on collagenase activity and MMP-8 and MMP-13 expression in the neointima. (A) Representative images of in situ collagenase activity (scale bar  = 100 µm) and quantification in the control and atorvastatin-treated groups (*p<0.05, n = 6). (B) Representative images of MMP-8 immunohistochemical labeling (scale bar  = 100 µm) and quantification in the control and atorvastatin-treated groups (*p<0.05, n = 6). (C) Representative images of MMP-13 immunohistochemical staining (scale bar  = 100 µm) and quantification in the control and atorvastatin-treated groups (*p<0.05, n = 6). Values are the mean ± SEM.

Because upregulated collagen content was observed in the atorvastatin-treated group, we measured neointimal collagenase activity. As shown in Figure 2A, a decreased collagenase activity/intima ratio was observed in the atorvastatin-treated group compared to control (21.6±1.3% *vs*. 30.3±2.5%, *p*<0.05). We also found downregulated MMP expression in LCCA tissue of atorvastatin-treated group. The respective ratios of fluorescence areas of MMP8 and MMP13 to intima were increased to 42.4±0.9% and 34.6±1.0% in the control group compared to 27.0±1.8% and 16.7±2.7% in the atorvastatin-treated group (*p*<0.05, Figure 2B, 2C and 2D). Furthermore, detection of MMPs and macrophages by double-immunofluorescence labeling revealed that MMP8 and MMP13 were primarily expressed in macrophages (Figure 2B and 2C).

### Atorvastatin Inhibits Expression of Neointimal Chemokines and their Receptors on Peripheral Blood Monocytes in ApoE−/− Mice

**Figure 3:**
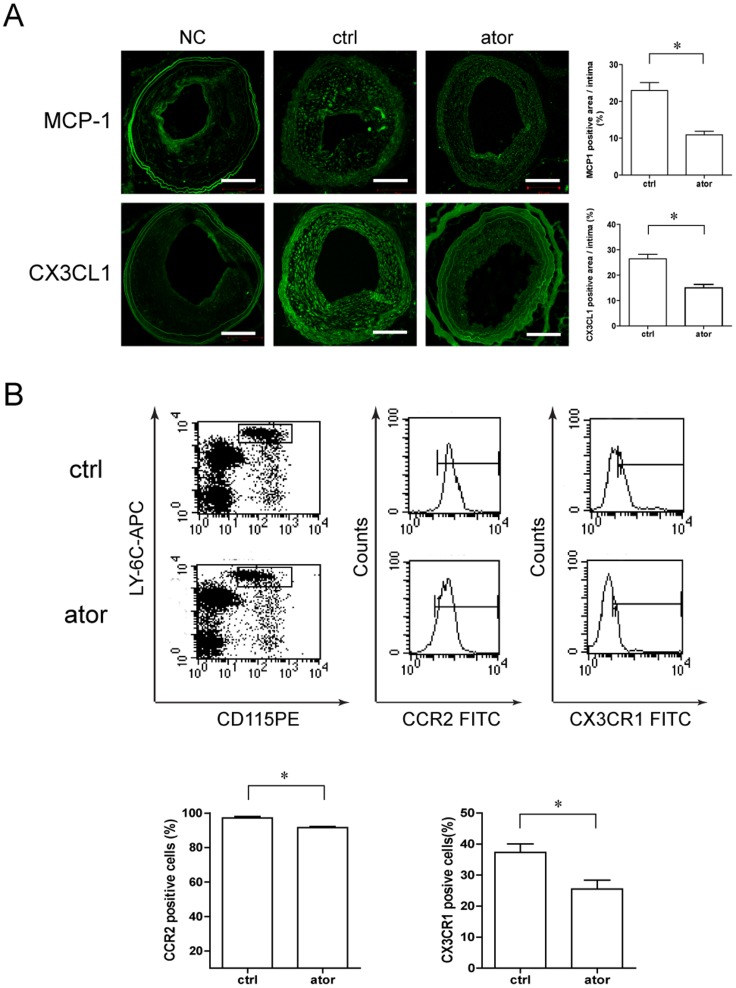
Effect of atorvastatin (10 mg/kg/d)on expression levels of intimal chemokines and their receptors e on peripheral blood monocytes in ApoE-/- mice. (A) Representative images of immunostaining for MCP-1 and CX3CL1 (scale bar  = 100 µm) and quantification in the control and atorvastatin-treated groups (*p<0.05, n = 6). (B) Representative FACS analysis for monocytes with double-immunofluorescent labeling for CCR2 and CX3CR1. Data represent the mean ± SEM of the percentage of marker-positive cells from six experiments. *p<0.05 compared with control.

Because we observed changes in the numbers of macrophages but not SMCs in atorvastatin-treated mice, we considered that improved plaque stability might explain the decrease in macrophage quantity compared to control mice. Then, we asked whether this resulted from atorvastatin treatment interrupting monocyte infiltration into atherosclerotic plaques. As expected from the intimal immunofluorescent labeling shown in Figure 3A, chemokine MCP-1 and CX3CL1 expression levels were both significantly decreased by atorvastatin (10.9±1.0% *vs*. 23.0±2.1% *p*<0.05 and 15.0±1.4% *vs*. 26.4±1.8% *p*<0.05, respectively). At the same time, mice peripheral blood monocytes identified as double-labeled for CD115 and Ly-6C antigens were analyzed by FACS. The expression levels of the corresponding chemokine receptors CCR2 and CX3CR1 were also inhibited in atorvastatin-treated mice (91.5±0.7% *vs*. 97.1±1.0% p<0.05 and 30.0±1.6% vs. 38.5±1.8% p<0.05, respectively, Figure 3B). And there was a significant association between reduced levels of neointimal chemokines and decreased macrophage quantity, lipid deposition, collagenase activity, as well as increased collagen content (p<0.05, see Table S2 in the File S1).

### Atorvastatin Decreases Plasma Levels of Inflammatory Markers in ApoE−/− Mice

**Figure 4:**
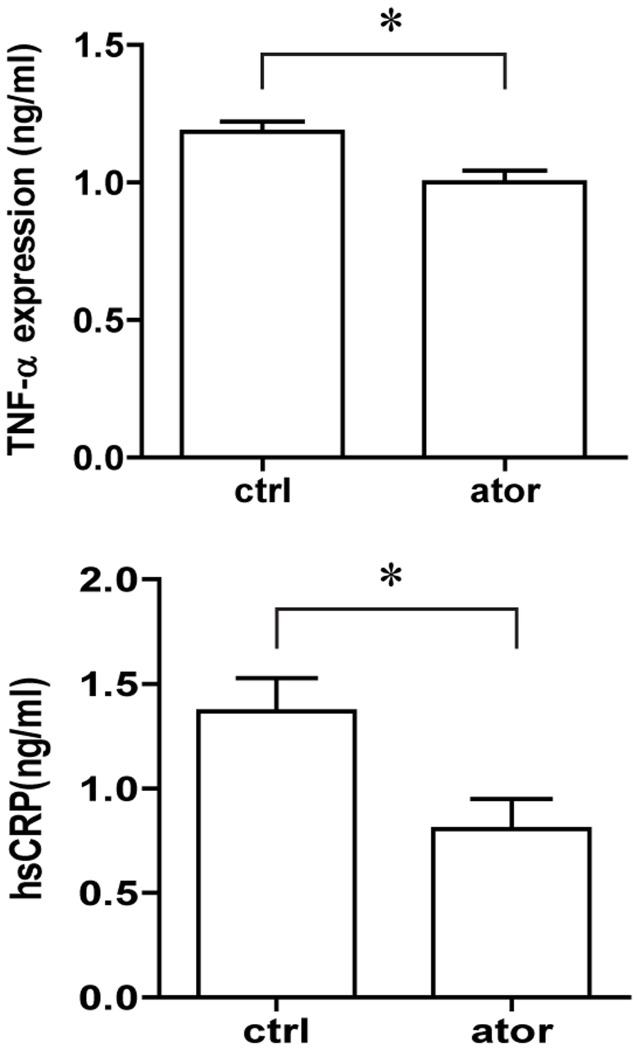
Effect of atorvastatin (10 mg/kg/d ) on plasma inflammatory markers TNF-α and CRP in ApoE-/- mice. Blood was collected 8 weeks after isosmotic saline or atorvastatin administration. Data represent the mean ± SEM of six ELISA experiments. *p<0.05 compared with control.

Finally, we verified the anti-inflammatory effect of atorvastatin by measuring inflammatory markers in plasma. As shown in Figure 4, the mean TNF-α level was significantly decreased in the atorvastatin-treated group compared to control (1.0±0.04 *vs*. 1.2±0.04 ng/ml, *p*<0.01). Plasma hsCRP levels showed a similar trend; a markedly reduced concentration was observed in the atorvastatin-treated group compared with the control group (0.81±0.14 *vs*. 1.37±0.16 ng/ml, *p*<0.05).

## Discussion

Atorvastatin is one of the most widely used HMG-CoA reductase inhibitors in lipid-lowering therapy; however, evidence is accumulating that atorvastatin is atheroprotective by lowering cholesterol levels and also by its pleiotropic biological effects [12]–[15]. In this study, we demonstrated that atorvastatin (10 mg/kg/d) treatment improved plaque stability by reducing inflammation and modulating chemokine and chemokine receptor expression without changing plasma cholesterol levels (see Table S1 in File S1).

We found that the number of vulnerable phenotype lesions was lower in atorvastatin-treated mice, but there was no difference in intima-to-media area ratio between the control and atorvastatin-treated groups. This indicates that although atherosclerotic plaque size was not reduced, lesion components might be altered by atorvastatin treatment [16]. Detailed analysis revealed that macrophages number and cholesterol deposition were both reduced in atorvastatin-treated mice. The plaque collagen content of atorvastatin-treated mice was higher, which was consistent with the improved lesion type, but the SMC numbers were not changed. These findings suggest that the improved plaque stability might be due to fewer macrophages in the intimal area. A reduction in subendothelial macrophages leads to less foam cell formation and cholesterol deposition, as well as less collagen proteolysis. Further experiments verified downregulated MMP expression levels and less macrophage-associated collagenase activity in atorvastatin-treated mice. Because vascular inflammation is in large part driven by changes in monocyte/macrophage numbers in the vessel wall [17], the anti-inflammatory effect of atorvastatin may partially be explained by a reduction in subendothelial macrophages.

Chemokines responsible for monocyte recruitment probably include CC chemokine ligand (CCL)-5, also known as RANTES; CCL-2, also known as MCP-1 and CX3CL1, also known as fractalkine in humans. Blocking experiments have suggested that inflammatory Ly-6C+ monocyte recruitment to atherosclerotic sites is reduced by 40% to 50% when CCR5, CCR2, or CX3CR1 is targeted. Therefore, we hypothesized that atorvastatin might interrupt monocyte recruitment into atherosclerotic plaques by regulating the expression of chemokines and/or their receptors. MCP-1 and its receptor CCR2, as well as CX3CL1 and its receptor CX3CR1, were reduced in the atorvastatin-treated group. CCR2 is expressed on the majority of blood monocytes, other leukocytes, and a subset of T cells, mainly responding to directed cell migration toward its primary ligand MCP1 [18]. In addition, CCR2-deficient animals ex decreased susceptibility to atherosclerosis and decreased intimal hyperplasia following arterial injury [19]–[20]. Similarly, CX3CR1 is expressed on monocytes, natural killer cells, a subset of T cells, and SMCs [21]–[22]. Its ligand CX3CL1, a membrane-bound chemokine, is increased in atherosclerosis [23]. The ability of this molecular pair to mediate leukocyte adhesion and migration under physiological flow conditions in vitro was first reported in 1998. A recent study showed that CX3CR1-deficient mice exhibit impaired macrophage and dendritic cell accumulation during vascular inflammation and are protected from intimal hyperplasia in an arterial injury model [24].

In conclusion, 10 mg/kg/d atorvastatin treatment inhibited unstable plaque development in ApoE-deficient mice independent of plasma cholesterol levels. The beneficial effect of atorvastatin is achieved via the inhibition of macrophage infiltration due to the modulated expression of chemokines and their receptors.

## Materials and Methods

### Mice

All animal work was performed according to the Shanghai Jiaotong University School of Medicine guidelines for the ethical care of animals. The protocol was approved by the Committee on the Ethics of Animal Experiments of the Shanghai JiaoTong University School of Medicine (Permit Number: [2012]-86). ApoE-knockout (ApoE−/−) mice on a C57BL/6 background were purchased from the Jackson Laboratory (Bar Harbor, ME, USA). At the age of 8 weeks, combined partial ligation of the left renal artery and left common carotid artery (LCCA) was performed under a dissecting microscope as previously described [11], and then sex-matched mice were randomly divided into two groups: control (n = 10) and atorvastatin (n = 12). Atorvastatin (Pfizer Ltd., New York, NY, USA) was dissolved in isosmotic saline and infused via a stomach tube at a dose of 10 mg/kg/day after surgery for 8 weeks. Controls were infused an equal volume of isosmotic saline. During the entire period, the mice had free access to water and food. Experimental mice were euthanized by cervical dislocation 8 weeks after the ligation surgery.

### Tissue Collection and Processing

All the mice were perfused with ice-cold normal saline, and each group was divided into two subgroups. In one subgroup, the bilateral carotid arteries were isolated, snap frozen in liquid nitrogen, and then stored at −80°C until use. The other half was perfused with 4% paraformaldehyde in 0.01 mol/L phosphate buffer (pH 7.4) under physiological pressure. Tissues were then fixed overnight for paraffin-embedding. Serial 4-µm cryosections were paraffin-embedded, and 5-µm sections were OCT-embedded, and each fragment of carotid artery from the distal bifurcation of carotid artery was sectioned every 200 µm over a 2-mm length.

### Morphometry

Cryosections (4-µm thick) were prepared from the dissected LCCA as described previously [11]. Virmani's criteria were used to classify lesion stage into stable or vulnerable plaque in hematoxylin and eosin (H&E)-stained sections. In brief, classes 1 and 2 are considered stable, whereas lesions in classes 3 to 6 are vulnerable plaques. A thin cap (<3 cell layers) accompanied by a large necrotic core (>40%) is defined as a thin-cap fibroatheroma lesion. Plaque rupture was defined as an area of fibrous cap disruption in which the overlying thrombus is in continuity with the underlying necrotic core. Intraplaque hemorrhage is defined as the deposition of blood products within the plaque that is not necessarily associated with rupture.

### Histology and Immunohistochemistry

To assess cholesteryl ester and collagen contents, slides were stained with Oil Red O and Sirius red, respectively, as previously described. The slides used in these experiments were selected based on their morphometric classification and were mostly lipid-laden in their serials. In brief, frozen sections used for lipid detection in atherosclerotic lesions were stained in a working solution of Oil Red O for 30 minutes and then rinsed in 60% isopropanol for 5 seconds. After washing thoroughly in phosphate-buffered saline (PBS), the slides were mounted with glycerin, and images were acquired. The collagen contents of the vascular intima were evaluated with Sirius red staining. Specimen sections were deparaffinized, rehydrated, incubated in 0.1% Sirius red in saturated picric acid for 60 min followed by 1% acetic acid for 30 min, counterstained in hematoxylin, differentiated in acid alcohol solution, rehydrated, and then mounted.

Slides from these two experiments were visualized under a bright-field microscope(Leica DM2500, Tokyo, Japan), and pictures of the entire slice were taken with identical exposure settings for all sections and analyzed using image analysis software (ImageJ, National Institutes of Health, Bethesda, MD, USA). The content of collagen was evaluated as percent of the vascular intima area.

Unfixed cryosections (5-µm) were incubated in matrix metalloproteinase (MMP) activity buffer containing 100 mM NaCl, 100 mM Tris–HCl (pH∶7.5), 10 mM CaCl2, 20 µM ZnCl, and 0.05% Brij 35 for 18 hours at 37°C to measure collagenase activity by in situ zymography. DQTM collagen type I (1 mg/ml, Molecular Probes/Invitrogen, Carlsbad, CA, USA) as a substrate was diluted in the buffer to a working concentration of 20 µg/ml. Negative controls were prepared with the collagen substrate solution including 1,10-phenanthroline (10 mmol/L, Sigma-Aldrich, St. Louis, MO, US). Analysis was performed by assessing the green fluorescent signal using an LSM 710 confocal microscope (Carl Zeiss, Oberkochen, Germany).

### Immunofluorescence

A segment of the LCCA containing atherosclerotic plaques was harvested and stored at –80°C. LCCA segments were fixed in cold paraformaldehyde for 10 min and serial cross-sections (5 µm) were triple-labeled with antibodies. Briefly, sections were blocked with 5% fetal calf serum (FCS) for 30 min and incubated with primary antibodies against MMP-8, MMP13, fractalkine (CX3CL1), (rabbit anti-mouse, dilution 1∶100, Abcam, Cambridge, UK) and monocyte chemotactic protein-1 (MCP1, rabbit anti-mouse, dilution 1∶100, Santa Cruz Biotechnology, Santa Cruz, CA, USA), CD68 (rat anti-mouse, dilution 1∶100, Millipore, Billerica, MA, USA), α-actin (donkey-anti-mouse, dilution 1∶100, Millipore) at 4°C overnight. Secondary antibodies labeled with magenta fluorescence (goat anti-rat, 647 nm, Invitrogen), red fluorescence (donkey-anti-mouse, 555 nm, Invitrogen), or green fluorescence (donkey-anti-rabbit, 488 nm, Invitrogen) were used (dilution 1∶300 in bovine serum albumin [BSA], Invitrogen) to visualize the primary antibodies. After a 60-min incubation, the nuclei were stained with DAPI (4',6-diamino-2-phenylindole, blue fluorescence), followed by digital image acquisition during confocal microscopy (Zeiss LSM 710).

### Cytokine and Inflammatory Marker Assays

Sandwich enzyme-linked immunosorbent assays (ELISAs) were used to detect plasma levels of C-reactive protein (CRP) (ALPCO, Salem, NH, USA) and TNF-α (eBioscience, San Diego, CA, USA) according to the manufacturers' instructions.

### Flow Cytometry Analysis of Blood Leukocytes

We collected 100-µl aliquots of tail blood into EDTA-containing tubes. Cells in some tubes were Fc-blocked with 1 µg of mouse IgG (105 cells) for 15 min at room temperature without excess washing. After blocking, cell suspensions were then labeled with several monocyte-surface markers (anti-CD115–PE, eBioscience, catalog #17-1152; and anti-Ly-6C–APC, eBioscience, catalog #12-5932) and anti-CCR2–FITC (B&D, catalog: FAB5538F) or anti-CX3CR1-FITC (catalog #FAB5825G, R&D Systems, Minneapolis, MN, USA) at 4°C for 30 min. After cells were lysed by the addition of 4 ml Whole Blood Lysing Reagent (catalog #FC002, R&D Systems), the cells were washed twice with ice-cold PBS followed by centrifugation at 300× g for 5 min. The pellets were resuspended by adding 150 µl PBS. Controls included cells incubated with fluorochrome-conjugated unspecific isotype Ab as needed. Cell-surface molecule expression measurement and cell sorting were performed on a FACSCalibur flow cytometer (BD Biosciences, Franklin Lakes, NJ, USA). Acquired fluorescence-activated cell sorting (FACS) data files were analyzed using the flow cytometry CellQuest software (BD Biosciences). Blood CD115+/Ly-6high cells defined as monocytes were further analyzed for CX3CR1 and CCR2 expression.

### Statistical Analysis

Values are expressed as mean ± standard error of the mean (SEM). Differences between groups were compared by two-tailed Student's t-tests. Differences in the classification and occurrence of adverse events were analyzed with X^2^ tests. P values <0.05 were considered significant.

## Supporting Information

File S1 **Supplemental materials.**
(DOC)
