# Supplementary material for: Atorvastatin Improves Plaque Stability in ApoE-Knockout Mice by Regulating Chemokines and Chemokine Receptors
Source: PLoS One. 2014 May 9;9(5):e97009. doi: 10.1371/journal.pone.0097009 (PMC4016207; doi:10.1371/journal.pone.0097009)
Supplement: File S1 — Supplemental materials. (DOC) [file pone.0097009.s001.doc]

**SUPPLEMENTAL MATERIAL**

Atorvastatin improves plaque stability in ApoE-knockout mice by regulating chemokines and chemokine receptors

Peng Nie ^a,1^, Dandan Li ^a,1^, Liuhua Hu ^a^, Shuxuan Jin ^a^, Ying Yu ^a^, Zhaohua Cai ^a^, Qin Shao ^a^, Jieyan Shen ^a^, Jing Yi ^b^, Hua Xiao ^a^, Linghong Shen ^a^,*, Ben He ^a^,*

*^a^ Department of Cardiology, Renji Hospital, School of Medicine, Shanghai Jiaotong University, Shanghai 200127, China;*

*^b^ Department of Cell Biology, Key Laboratory of the Education Ministry for Cell Differentiation and Apoptosis, Institutes of Medical Sciencies, School of Medicine, Shanghai Jiaotong University, Shanghai 200025, China*

Correspondence to:

Ben He ([rjheben@126.com](mailto:rjheben@126.com)) and Linghong Shen ([rjshenlinghong@126.com](mailto:rjshenlinghong@126.com))

Tel: +86 21 58752345; fax: +86 21 6838 3609.

^1^ These authors contributed equally to this work.

**Supplemental materials and methods**

**Determination of Plasma Lipid Profile**

At the end of study, blood was collected in heparinized tubes from anesthetized mice by left ventricular puncture. Plasma was obtained by centrifugation (5,000 rpm) at 4°C for 10 min and stored at -80°C. Plasma concentrations of triglyceride and total cholesterol were measured on a Hitachi 7180 autoanalyzer (Hitachi High-Technologies Corp., Japan) according to the manufacturer’s instruction.

**Table S1.** Plasma cholesterol and triglyceride levels. Data represent the means±SD.

| Mice | n | TC  (mmol/L) | TG  (mmol/L) |
| --- | --- | --- | --- |
| control | 10 | 11.96±2.27 | 1.07±0.39 |
| atorvastatin | 11 | 11.74±1.44 | 1.08±0.43 |

**Correlation between hemokines/receptors and macrophage/SMC/oil red O/collagen content/MMPs was analyzed by Pearson Correlation Coefficient.**

**Table S2.**

| Coefficient | CD68 | actin | Oil red | Collagen | MMP-8 | MMP-13 |
| --- | --- | --- | --- | --- | --- | --- |
| CX3CL1 | 0.61 * | -0.28 | 0.85 * | -0.76 * | 0.68 * | 0.74 * |
| MCP-1 | 0.68 * | -0.01 | 0.69 * | -0.60 | 0.38 | 0.86 * |
| CCR2 | 0.70 | 0.07 | 0.76 | -0.58 | 0.53 | 0.89 * |
| CX3CR1 | 0.98 * | -0.23 | 0.82 * | -0.79* | 0.82 * | 0.81 * |

* *p* < 0.05.
